# Individual characteristics influencing the general population’s level of knowledge of end-of-life practices: a cross-sectional study

**DOI:** 10.1177/26323524241312922

**Published:** 2025-01-27

**Authors:** Diane Tapp, Gina Bravo, Catherine Filion, Vincent Couture, Sophie Dupéré, Marianne Beaulieu, Audrey Chouinard, Pauline Roos, Marie-Pierre Gagnon, Anouk Bérubé, Ariane Plaisance

**Affiliations:** Faculté des Sciences Infirmières, Université Laval, Pavillon Ferdinand-Vandry, 1050, Avenue de la Médecine, Québec, QC G1V 0A6, Canada; Centre de Recherche du CHU de Québec, Université Laval, 1401, 18e rue, Québec, QC G1J 1Z4, Canada; Faculté de Médecine et Sciences de la Santé, Université de Sherbrooke, Sherbrooke, QC, Canada; Faculté des Sciences Infirmières, Université Laval, Québec, QC, Canada; Faculté des Sciences Infirmières, Université Laval, Québec, QC, Canada; Centre de Recherche du CHU de Québec, Université Laval, Québec, QC, Canada; Faculté des Sciences Infirmières, Université Laval, Québec, QC, Canada; Faculté des Sciences Infirmières, Université Laval, Québec, QC, Canada; Centre de Recherche du CHUM, Montréal, QC, Canada; Haute École Arc Santé, Delémont, Switzerland; Faculté des Sciences Infirmières, Université Laval, Québec, QC, Canada; Centre de Recherche du CHU de Québec, Université Laval, Québec, QC, Canada; Faculté des Sciences Infirmières, Université Laval, Québec, QC, Canada; Faculté des Sciences Infirmières, Université Laval, Québec, QC, Canada

**Keywords:** advance care planning, caregiving, death literacy, end-of-life, Medical Aid in Dying, palliative care, socioeconomic status

## Abstract

**Background:**

Informed end-of-life decision-making requires a high level of death literacy. We still know little about the general population’s level of knowledge and its determinants.

**Aim:**

To assess knowledge of the general population regarding the legal status and definitions of various end-of-life practices, and to compare the level of knowledge according to individual characteristics known to influence death literacy.

**Design:**

A self-administered questionnaire featuring two evolving vignettes was used to assess participants’ knowledge relating to the legal status of various end-of-life practices and whether these practices are Medical Aid in Dying (MAiD), which is legal in Canada. The questionnaire also assessed participants’ individual characteristics such as their experience as caregivers for someone who received palliative care, their perception of health, and their financial situation.

**Setting/participants:**

Participants were community-based community-based Canadian adults able to read French or English.

**Results:**

In total, 27% of the participants associated the description of care withholding with MAiD, 39% incorrectly associated the description of continuous palliative sedation with MAiD, and 34% incorrectly indicated that the described intervention was illegal. Having cared for someone who received palliative care, at a younger age, a higher level of education, and having participated in advance care planning were associated with better knowledge regarding end-of-life practices.

**Conclusion:**

Gaps in knowledge about end-of-life practices exist in the general population, they are associated with different individual characteristics and may limit citizens’ capacity to engage in informed end-of-life decision-making. Community-based interventions adapted to different audiences are essential to ensure a quality end-of-life for all.

## Background

In Western Europe and North America, death is increasingly preceded by numerous clinical decisions, whereas most people die in medical institutions.^1^ Any clinical decision must be preceded by an informed consent. Five elements of information are essential for informed consent: a discussion of the patient’s health condition, a description of the procedure itself, as well as a discussion of the procedure’s benefits, alternatives, and risks.^2^ To fully engage in end-of-life decision-making, one needs realistic and accurate information about end-of-life options and likely outcomes.^3^ This implies that the decision maker has a high level of death literacy. Similar to the concept of “health literacy,”^4^ “death literacy” is defined as a set of knowledge and skills that make it possible to access, understand, and act upon the end-of-life journey and end-of-life options.^5^ One’s level factors include culture, religion, education, age, and personal experiences with illness, death, and grief. Regarding the importance of personal experience, caring for someone at the end of their lives provides a deep personal connection to death and dying and can serve as a catalyst for developing death literacy.^5^ For instance, caregivers of people with Alzheimer’s or a related disease must be involved in the decision-making process determining which end-of-life practices are best for the individual since patients who have reached the end stage of their illness are no longer capable of consenting to treatment.^6^

The *Act respecting end-of-life care*,^7^ voted in 2015 in the Canadian province of Quebec, allows physicians to end the life of a competent person at their request. This intervention, elsewhere named euthanasia, is called Medical Aid in Dying (MAiD) in Quebec (as well as in the rest of Canada). It includes: being at least 18 years of age, suffering from a serious and incurable disease at an advanced state of irreversible decline in capability, and experiencing constant and unbearable suffering that cannot be relieved in a way they judge to be acceptable.^7^ Since October 30, 2024, individuals have been able to formulate advance requests for medical assistance in dying to be implemented once they become incapacitated.

Confusion between the various end-of-life practices has been documented in many studies,^8–11^ including in Quebec.^12,13^ Before the legalization of MAiD, individuals’ confusion between treatment withdrawal and the administration of morphine was associated with higher support for its legalization.^9^ Also, before the legalization of MAiD, a survey targeting healthcare professionals showed that 46% of the participants wrongly believed that it was not legal to withdraw a potentially life-prolonging treatment at the patient’s request.^13,14^ Analysis of the 63 submissions on the bill that led to the *Act respecting end-of-life care*, revealed that the less the authors paid attention to the section on continuous palliative sedation, the more likely they were to demonstrate a pro-MAiD attitude.^15^

Although the act to amend the Act respecting end-of-life care and other legislative arrangements was adopted on June 7, 2023,^16^ we know little about the knowledge of the general population regarding various end-of-life practices. Our objective was therefore to assess Quebec’s general population’s knowledge of various end-of-life practices and to determine whether individual characteristics such as perceived health, perceived financial situation, and caregiving experiences are associated with the level of knowledge.

## Methods

### Design

A bilingual (French and English) community-based questionnaire study was carried out in the province of Quebec, Canada. This article reports descriptive, cross-sectional, quantitative population survey data addressing individual characteristics influencing the general population’s level of knowledge of end-of-life practices.

### Participants

Participants were community-based adults (18 years of age or older) from Quebec able to read French or English.

### Recruitment and data collection

With the help of a social media professional, we developed a recruitment strategy using diverse means (social media, networking, and posters) adapted to the specificities of four subgroups. Indeed, to address our objective, we intentionally targeted: (1) people who have a negative perception of their health; (2) people who have a negative perception of their financial situation; (3) people who cared for someone receiving palliative care; and (4) people who cared for someone with dementia or a related disease receiving palliative care. Participants self-screened their eligibility and then completed the questionnaire online on the REDCap (Research Electronic Data Capture) platform, anonymously. REDCap is a secure web application for building and managing online surveys and databases. By completing the questionnaire, participants gave the research team permission to collect and analyze the information they provided. Completed questionnaires were automatically assigned an ID number. Data were stored on the research center’s secure server. The survey ran from October 24, 2019, to February 1, 2020.

The planned sample size was estimated based on the use of multivariable ordinal regression analyses. According to the literature, 30 observations per subgroup are needed.^17^ Hence, we aimed to recruit 120 participants.

The questionnaire was adapted from one used in other studies conducted in Quebec.^18,19^ The self-administered questionnaire introduced two evolving vignettes leading to the introduction of MAiD and other end-of-life practices. The following definitions were provided at the start of the questionnaire:

- MAiD is defined as “care consisting in the administration by a physician of medications or substances to an end-of-life patient, at the patient’s request, to relieve their suffering by hastening death”^7^;
- Continuous palliative sedation is inducing unconsciousness in patients with a poor prognosis as they approach death and until death^20^;
- Treatment withholding and withdrawing is forgoing life-sustaining treatments or ceasing such treatment, thus leading to the patient’s natural death^21^;
- Administration of high doses of sedatives in a palliative emergency consists of administrating an opioid, an anticholinergic, and a sedative to induce sedation and rapidly decrease the acute symptoms of a patient at the end of life.^22^

The first vignette involved a terminal cancer patient who could consent to his healthcare choices (Supplemental Appendix 1), while the second was about a patient who had lost her capacity to consent due to cognitive impairment (Supplemental Appendix 2). The questionnaire was pre-tested with respect to the applicable best practices.^23^ The questionnaire is available upon request to the corresponding author.

### Measures

#### Sociodemographics

Participants’ age, gender, level of education and religiosity, involvement in advance care planning, perceived health and financial situation, and experience caring for someone receiving palliative care or someone who has Alzheimer disease or a related disease (e.g., Parkinson) receiving palliative care were collected. Having acted as the caregiver of someone with Alzheimer’s or a related disease might contribute to increasing one’s knowledge regarding end-of-life practices in this specific context.

#### Knowledge of end-of-life practices

The first section aimed to assess participants’ knowledge and attitudes concerning five practices: An order protocol for distress, treatment withholding, assisted suicide, MAiD, and continuous palliative sedation. The two latter practices were introduced in both vignettes. For each vignette, questions about knowledge centered around whether the described practice corresponded with MAiD and whether it was legal in the province of Quebec at the time of the study (“yes,” “no,” “I do not know”).

Answers to each of the knowledge questions were coded as 0 (incorrect) or 1 (correct), and “I do not know” was coded as 0. Then, the codes were summed to obtain a total knowledge score ranging from 0 (lack of knowledge) to 14 (high knowledge).

### Data analysis

Analyses were performed using SPSS v. 27.^24^ Given the high number of participants and the very small number of missing data, no process was carried out to deal with missing data. Incomplete questionnaires were not considered in the regression analyses. We used descriptive statistics to summarize sociodemographic data (mean, standard deviation, counts, and percentages).

We conducted an ordinal regression analysis to simultaneously examine the associations of sociodemographic characteristics with the overall knowledge score. Assumptions underlying the analytic approach were tested and confirmed. To meet the proportional odds model’s assumption, we performed a full likelihood ratio test comparing its fit to a model with varying location parameters, revealing that the assumption of proportional odds was met: χ^2^ (14) = 19.7, *p* = 0.141. No multicollinearity was found between predictors and, hence, all candidate predictors were included in the multivariable model. Results were interpreted at the 0.05 significance level.

## Results

### Sociodemographic characteristics of participants

One thousand two hundred eight (1208) people opened the questionnaire and nine hundred forty-six (946) ended it (Table 1). Eight hundred and seventy-two (872) answered all the questions and were included in the sociodemographic data. All (100%) were living in the province of Quebec and age varied from 19 to 90 years (mean ± standard deviation (SD): 56.7 ± 13.5). Most were women (92.3%). Nearly half had a university degree (44.7%), but more than a quarter (28.6%) had completed a high school diploma or less. Most participants (77.6%) had a very good perception of their financial situation. Nearly half had completed some form of advance care planning (44.2%). Most participants cared for someone receiving palliative care (73.4%), but less than half cared for someone with Alzheimer disease or a related disease receiving palliative care (37.7%).

**Table 1.** Sociodemographic characteristics of participants (*N* = 872).

Characteristic	Value
Age, mean ± SD (range)	56.7 ± 13.5 (19–90)
Gender, *n* (%)	
Women Men Other	805 (92.3) 66 (7.6) 1 (0.1)
Level of education, *n* (%)	
High school diploma or less College degree University degree	249 (28.6) 233 (26.7) 390 (44.7)
Importance of religion, *n* (%)	
Not/somewhat important Moderately important Very/extremely important	569 (65.3) 217 (24.9) 86 (9.9)
Completed some form of advance care planning, *n* (%)	385 (44.2)
Perception of health, *n* (%)	
Not good Good Very good/excellent	102 (11.7) 264 (30.3) 506 (58.0)
Perception of financial situation, *n* (%)	
Not good Good Very good/excellent	35 (4.0) 160 (18.3) 677 (77.6)
Cared for someone receiving palliative care, *n* (%)	640 (73.4)
Cared for someone with Alzheimer disease or a related disease (e.g., Parkinson) receiving palliative care, *n* (%)	329 (37.7)

### Participants’ knowledge of end-of-life practices

Mean knowledge score was 9.5 (SD 3.6) out of a potential total of 14 (Table 2).

**Table 2.** Participants’ knowledge of end-of-life practices (*N* = 946).

Items	Correct answer	Accurate responses, *n* (%)
Description of palliative continuous sedation (scenario 1)		
Is the described intervention legal?	Yes	623 (65.9)
Is the described intervention MAiD?	No	579 (61.2)
Description of MAiD (scenario 1)		
Is the described intervention legal?	Yes	635 (67.1)
Is the described intervention MAiD?	Yes	767 (81.1)
Description of assisted suicide (scenario 1)		
Is the described intervention legal?	No	745 (78.8)
Is the described intervention MAiD?	No	617 (65.2)
Description of the use of high doses of sedatives in a palliative emergency (scenario 1)		
Is the described intervention legal?	Yes	527 (55.7)
Is the described intervention MAiD?	No	718 (75.9)
Description of the enactment of an advance request for MAiD (scenario 2)		
Is the described intervention legal?	No	693 (73.3)
Is the described intervention MAiD?	No	493 (52.1)
Description of treatment withholding (scenario 2)		
Is the described intervention legal?	Yes	706 (74.6)
Is the described intervention MAiD?	No	688 (72.7)
Description of palliative continuous sedation (scenario 2)		
Is the described intervention legal?	Yes	604 (63.8)
Is the described intervention MAiD?	No	586 (61.9)

MAiD, Medical Aid in Dying.

The Pearson goodness-of-fit test revealed that the model fits the observed data well: χ^2^ (1526) = 1648.4, *p* = 0.113. The final model outperformed the intercept-only model in terms of statistical significance, χ^2^ (14) = 114.6, *p* = 0.001.

### Influence of individual characteristics on the level of knowledge

#### Caregiving experiences

Having cared for someone receiving palliative care was positively associated with knowledge of end-of-life practices (odds ratio (OR) = 1.63; 95% confidence interval (CI): 1.22–2.20, *p* = 0.001). Having cared for someone with Alzheimer’s disease or a related disease receiving palliative care showed a similar positive association (OR = 1.42; 95% CI: 1.09–1.86, *p* = 0.01).

#### Effects of other variables on knowledge

Age (expressed in years) was negatively associated with better knowledge (OR = 0.98; 95% CI: 0.97–0.99, *p* < 0.001; Table 3). Lesser knowledge about end-of-life practices was found among less educated participants (OR = 0.38; 95% CI: 0.27–0.52, *p* < 0.001). People having completed some form of advance care planning showed better knowledge (OR = 1.35, 95% CI: 1.04–1.76, *p* = 0.025). Importance of religion (*p* = 0.29), perception of health (*p* = 0.70), gender (*p* = 0.86), and self-perceived financial situation (*p* = 0.71) were not associated with knowledge.

**Table 3.** Results of the ordinal multiple regression.

Variables	*B* coefficient	CI	SE *B*
Age	**−0.02**	−0.03 to −0.01	0.01
Gender (women)	0.33	−3.31 to 3.98	1.86
Level of education (high school diploma or less)	**−0.98**	−1.30 to −0.66	0.16
Importance of religion (not)	0.24	−0.20 to 0.67	0.22
Advance care planning (yes)	**0.30**	0.04 to 0.57	0.13
Perception of health (not good)	−0.83	−0.50 to 0.34	0.21
Perception of financial wealth (very good)	−0.13	−0.80 to 0.54	0.34
Caregiving experience (not with dementia)	**0.49**	0.20 to 0.79	0.15
Caregiving experience (with dementia)	**0.35**	0.08 to 0.62	0.14

Significant correlations (*p* < 0.05) are in bold.

*B*, standardized regression coefficient; CI, confidence interval; Model, stepwise model in SPSS Statistics; SE *B*, standard error of the coefficient.

### Discussion

#### Key findings

We conducted a questionnaire study to identify individual characteristics influencing the general population’s level of knowledge regarding different end-of-life practices, including MAiD, continuous palliative sedation, treatment withholding, and the administration of high doses of sedatives in a palliative emergency.

The average result of participants’ knowledge of end-of-life practices indicates, overall, that the population has moderate to acceptable knowledge. Despite this result, various points appear problematic regarding the level of knowledge of the population. For many participants, the results show a misunderstanding regarding general knowledge of MAiD. Around one-third of the sample wrongly answered that the intervention corresponding to the description of MAiD was illegal. Approximately 27% of participants associated the description of treatment withholding with MAiD. These results are similar to the findings of other studies. According to a study by Marcoux, Mishara, and Durand conducted before the legalization of MAiD and among a representative sample of the general population in Quebec, even though 70% of respondents supported legalizing euthanasia (now MAiD), the majority were unable to correctly define what constitutes euthanasia, frequently believing that this referred to treatment withholding or withdrawing.^25^

We also observed a lack of knowledge on continuous palliative sedation. Around 39% of the sample incorrectly associated the description of continuous palliative sedation with MAiD, and 34% wrongly indicated that the described intervention was illegal. Continuous palliative sedation is an end-of-life practice that involves generating a state of unconsciousness to relieve severe and refractory symptoms in patients nearing death.^20^ The confusion between continuous palliative sedation and MAiD is widespread. According to the results of a study that involved clinicians in Flanders (Belgium), Oregon (USA), and Quebec (Canada), the relationship between continuous palliative sedation and euthanasia is frequently viewed as fluid and intricate, which contrasts with the laws, ethical standards, and clinical recommendations that are currently in place.^26^ When considering the implementation of advance requests for MAiD, it is crucial to have strict guidelines to make sure that those formulating such requests are asking for drugs to end their life, and not for medical measures aimed at ensuring comfort at the end of life or at withholding or withdrawing treatment.

Our findings demonstrated that awareness of various end-of-life practices was significantly and positively influenced by personal experience as caregivers, younger age, education level, and participation in advance care planning. These findings are comparable to those of an international study that quantified perceptions of and attitudes toward palliative care in a community-based sample and identified individual characteristics influencing perceptions and attitudes.^27^ Participants who had experience caring for someone with a serious illness, and those with a better understanding of palliative care were more likely to have more favorable attitudes toward the practice.^27^ Some studies have shown that younger adults prefer to be more involved in treatment decisions compared to older adults^28^ and that older adults have more misconceptions of palliative care,^29^ which is consistent with the literacy data in health where more than 60% of adults do not have a sufficient degree of literacy allowing them to adequately take care of their health and this percentage reaches 95% among people aged over 65.^11,30^ Information and community education initiatives related to palliative care and end-of-life practices should be developed. These initiatives must be tailored to address the specific needs of diverse populations.^31^

### What this study adds?

Advance requests for MAiD are already legal in some circumstances in Quebec, Belgium, Colombia, Luxembourg, and the Netherlands.^32^ Advance requests for MAiD raise moral, ethical, clinical, and legal challenges.^33–35^ Our results show that there is still confusion between care withholding, continuous palliative sedation, and MAiD. This study underscores how important it is to have strict rules to make sure that a person who makes an advance request for MAiD knows that they are asking for drugs to end their life, not for medical measures aimed at comfort at the end of life or for care withholding or withdrawal. In light of our results and the complexified set of knowledge needed to face dying and death, community-based interventions, adapted to different audiences and realities of the population concerned, are essential to enhance the knowledge of the general population regarding various end-of-life options and how to access them.^36^ For example, interventions targeting adolescents or young adults will differ in both content and format from those designed for older or very elderly individuals.^37^ Lack of knowledge about palliative options can lead people to have myths and misconceptions such as the use of injections to hasten death^38^ and associate palliative care with last resort care reserved only for the very end of life.^39^ To our knowledge, this is the first study in Quebec assessing the knowledge of the general population regarding MAiD and other end-of-life practices since the *Act respecting end-of-life care* came into effect in December 2015. This study is timely, and its results will contribute to current and future social debates and law evolutions.

### Study limitations

A self-selected community-based sample was used in this investigation. Even though we aimed to recruit an underrepresented sample of the population and used various recruitment strategies to achieve this objective, the sample was largely comprised of women with a high level of education. It is possible that there was a systematic bias among the respondents, such that those who have more knowledge and interest in end-of-life practices may have been more likely to volunteer to participate. For this reason, we can hypothesize that the confusion and lack of knowledge of the general population may still be far more important than what the present study suggests. Another significant limitation is that the data were collected before the pandemic and had not yet been analyzed. It is possible that the results no longer accurately reflect the current situation, given societal and legal developments. In this regard, further studies are necessary to account for these potential changes.

## Conclusion

Our findings confirm that knowledge is positively impacted by previous experiences, a younger age, a higher level of education, and having completed any kind of advanced care planning. We also found that participants conflated MAiD with other end-of-life practices, especially continuous palliative sedation and that significant proportions of participants were unaware of the legal status of MAiD and of other end-of-life practices. Future analyses exploring the influence of knowledge on attitudes toward end-of-life practices for different clientele (cancer vs dementia and related diseases) are needed. In view of our findings and the complicated body of knowledge required to deal modern-day dying and death, community-based interventions tailored to various audiences are crucial to raising the general public’s awareness of available end-of-life options and ways to access them.

## Supplemental Material

sj-docx-1-pcr-10.1177_26323524241312922 – Supplemental material for Individual characteristics influencing the general population’s level of knowledge of end-of-life practices: a cross-sectional study Supplemental material, sj-docx-1-pcr-10.1177_26323524241312922 for Individual characteristics influencing the general population’s level of knowledge of end-of-life practices: a cross-sectional study by Diane Tapp, Gina Bravo, Catherine Filion, Vincent Couture, Sophie Dupéré, Marianne Beaulieu, Audrey Chouinard, Pauline Roos, Marie-Pierre Gagnon, Anouk Bérubé and Ariane Plaisance in Palliative Care and Social Practice

## Declarations

**Ethics approval and consent to participate:** This multicenter project was approved by the Quebec Heart and Lung Institute—Université Laval Research Center’s ethics board (MP-10-2019-3237, 21783), as well as those of other partner institutions, on June 14, 2019.

**Consent for publication:** All authors approved the final version of the article to be published and take public responsibility for appropriate portions of the content.

**Availability of data and materials:** Not applicable.
