# Supplementary material for: Individual characteristics influencing the general population’s level of knowledge of end-of-life practices: a cross-sectional study
Source: Palliat Care Soc Pract. 2025 Jan 27;19:26323524241312922. doi: 10.1177/26323524241312922 (PMC11770702; doi:10.1177/26323524241312922)

**Supplementary file**

**Appendix 1.** A patient with cancer.


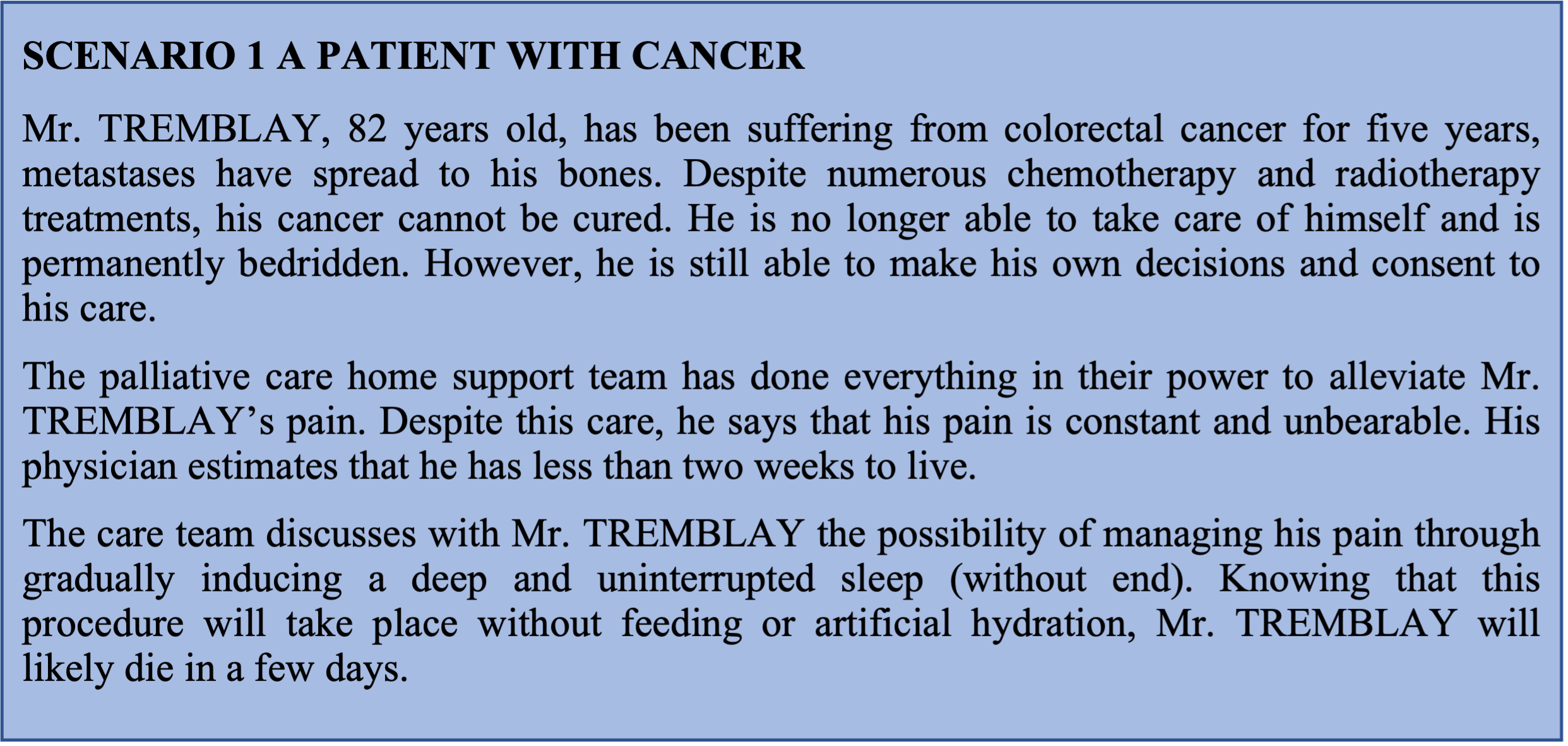


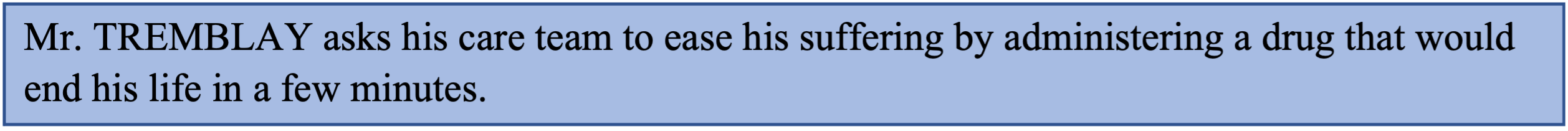


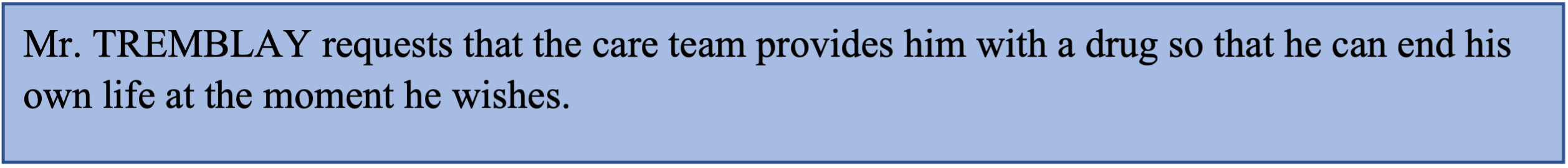


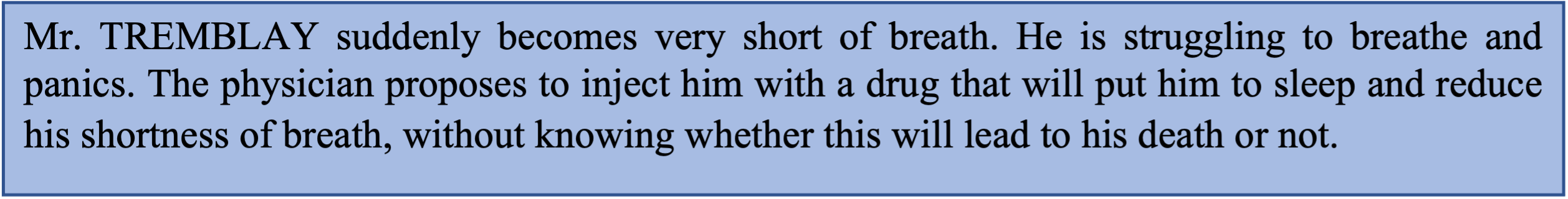


**Appendix 2.** A patient with Alzheimer’s disease.


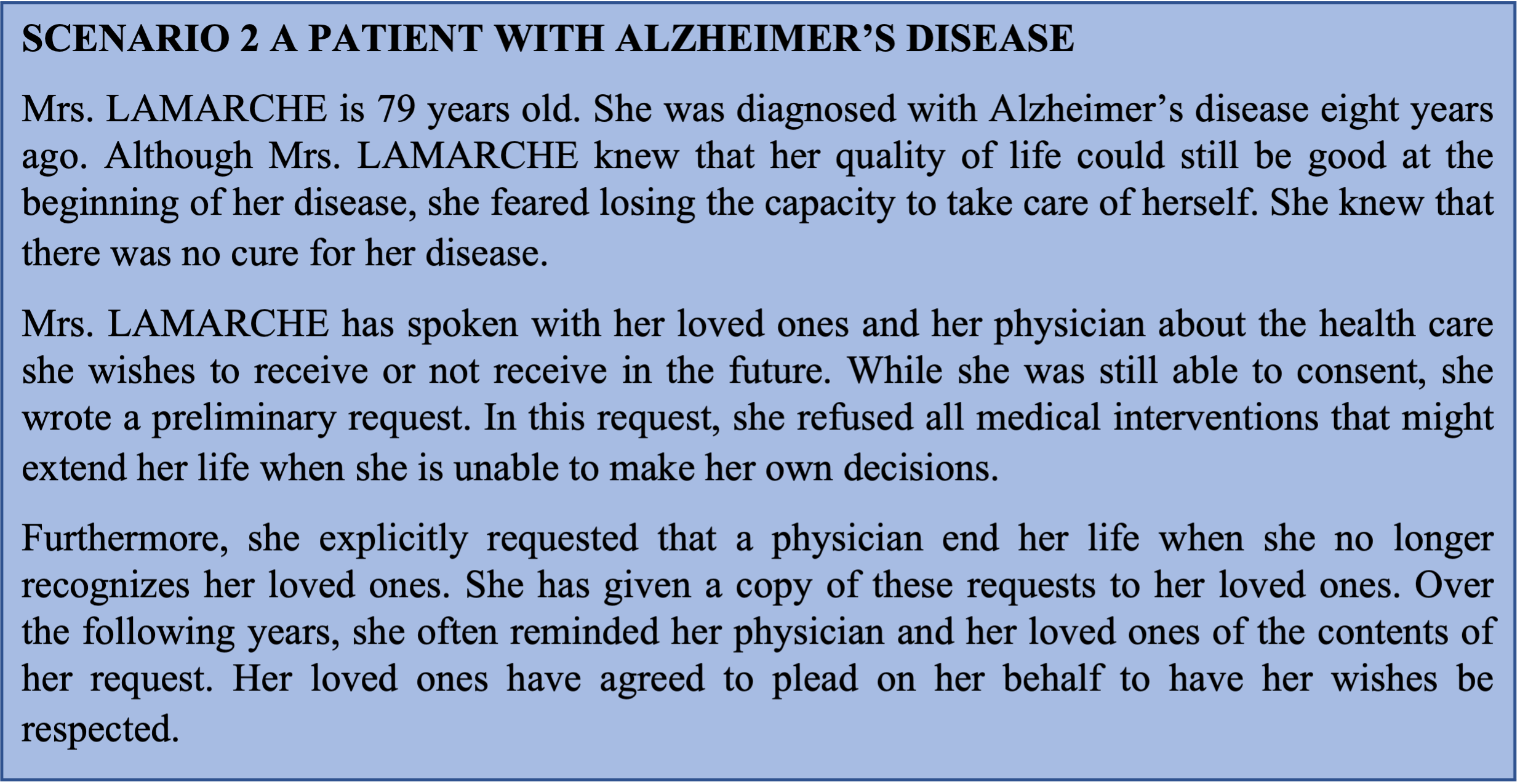


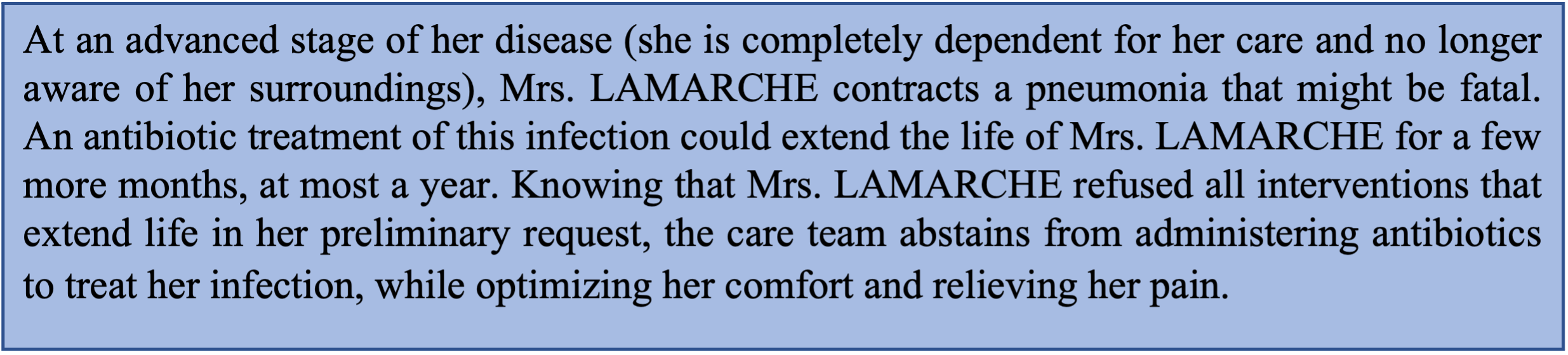


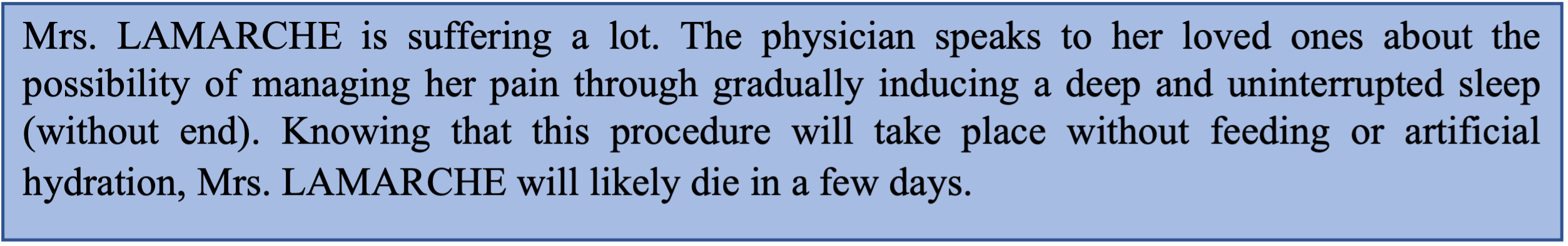

Supplement: sj-docx-1-pcr-10.1177_26323524241312922 – Supplemental material for Individual characteristics influencing the general population’s level of knowledge of end-of-life practices: a cross-sectional study [file sj-docx-1-pcr-10.1177_26323524241312922.docx]
